# Spike-Wave Seizures, NREM Sleep and Micro-Arousals in WAG/Rij Rats with Genetic Predisposition to Absence Epilepsy: Developmental Aspects

**DOI:** 10.3390/life12040576

**Published:** 2022-04-12

**Authors:** Maxim Zhuravlev, Anastasiya Runnova, Kirill Smirnov, Evgenia Sitnikova

**Affiliations:** 1 National Medical Research Center for Therapy and Preventive Medicine, Petroverigskiy Pereulok, 10(3), 101990 Moscow, Russia; runnova.ae@staff.sgmu.ru; 2 Institute of the Higher Nervous Activity and Neurophysiology of Russian Academy of Sciences, Butlerova Str., 5A, 117485 Moscow, Russia; kirillsmirnov@ihna.ru (K.S.); eu.sitnikova@ihna.ru (E.S.)

**Keywords:** animal model, absence epilepsy, automatic identification, EEG biomarkers, behavioral states, sleep fragmentation

## Abstract

The current study was done in Wistar Albino Glaxo Rijswijk (WAG/Rij) rats, which are genetically prone to develop spontaneous spike-wave discharges (SWDs) and are widely used as a genetic model of absence epilepsy. Here, we examined functional links between sleep and spike-wave epilepsy in aging WAG/Rij rats using advanced techniques of EEG analysis. SWDs, periods of NREM sleep and micro-arousals were automatically detected in three-channel epidural EEG recorded in freely moving WAG/Rij rats consequently at the age 5, 7 and 9 months. We characterized the developmental profile of spike-wave epilepsy in drug-naïve WAG/Rij rats and defined three epi-phenotypes—severe, mild and minor epilepsy. Age-related changes of SWDs were associated with changes in NREM sleep. Several signs of NREM sleep fragmentation were defined in epileptic WAG/Rij rats. It seems that spike-wave epilepsy per se promotes micro-arousals during NREM sleep. However, subjects with a higher number of micro-arousals (and NREM sleep episodes) at the age of 5 months were characterized by a reduction of SWDs between 5 and 7 months of age.

## 1. Introduction

Epilepsy is often accompanied by impairment of consciousness and/or disturbances of sleep-waking patterns. Typical absence seizures, such as childhood absence epilepsy and juvenile absence epilepsy are clinically manifested as sudden and brief (usually less than 10 s) lapses of consciousness [1]. Typical absence seizures have an electroencephalographic hallmark—bilaterally synchronous spike-wave discharges that occur during passive immobility, and light stages of non-rapid eye movement sleep (NREM) [2]. Normal sleep circuitry “can be hijacked to generate aberrant oscillatory network activity” [3] as a result of pathological processes in the thalamocortical neuronal network described in a large number of papers [4,5,6,7].

Absence epilepsy is known to affect the homeostatic regulation of slow-wave sleep (for Refs see [8,9]). “There seems to exist a critical zone of vigilance level (while falling asleep, during arousal from sleep, between NREM sleep, wakefulness and REM sleep) where SWDs are more likely to occur” [10]. On the one hand, SWDs are initiated by mechanisms responsible for sleep induction. On the other hand, the occurrence of SWDs destroys normal sleep/wake patterns. Patients with absence epilepsy show a decrease in REM, slow-wave sleep, and total sleep time [11]. Anti-absence medication itself might affect sleep-waking mechanisms, and sleep-related effects might vary in different cohorts of patients [12,13,14,15]. The use of drug-naïve animals as a reliable model can answer the question of the relationship between spike-wave discharges and sleep disturbances. The current study was done in Wistar Albino Glaxo Rijswijk (WAG/Rij) rats, which are genetically prone to develop spontaneous SWDs. The WAG/Rij rats model is well characterized as having construct, face and predictive validities widely accepted as a genetic model of absence epilepsy [16,17,18,19,20].

There is a close relationship between the level of vigilance and expression of spike-wave paroxysms [21]. In particular, SWDs in WAG/Rij rats predominantly occur during passive wakefulness (the state immobility) and light slow-wave sleep [16,22]. Absence epilepsy is a disorder of the corticothalamic system that is functionally linked to the initiation of NREM sleep [23]. NREM sleep is characterized by complex internal dynamics including a cyclic alternating pattern (CAP), which consists of phases of increased (phase A) and decreased (phase B) activation [24]. It was shown that SWDs are modulated by sleep microstructure, showing the greatest number of epileptic events during phase A [25,26]. To our knowledge, CAP has not been described in rodents, but similar cycles of sigma power (10–15 Hz) were found on a 50 s time scale during NREM in humans and mice [27]. This ~50 s sigma periodicity during non-REM sleep (corresponding to a 0.02 Hz oscillation) in mice might be somewhat similar to CAP in humans, but this pattern has not been shown in rats yet. The relationship between ultraslow periodicity with CAP has not been established, and further studies are needed in relation to cyclic patterns of the sleep microstructure in humans and rats.

It is known that young WAG/Rij rats do not express spike-wave seizures before the age of 2–3 months [28], and then the number and duration of SWDs gradually increase and reach a maximum at the age of 7–9 months [16,29,30]. The age-related increase of spike-wave epilepsy in WAG/Rij rats is known to be associated with some changes of the sleep/wake cycle as reviewed in [31]. Recently, we found some indirect evidence that spike-wave epilepsy in 9–11-month-old WAG/Rij rats is associated with sleep disruption and NREM sleep fragmentation [32]. In the current report, we further examine functional links between sleep and spike-wave epilepsy in aging WAG/Rij rats using advanced techniques of EEG analysis. In particular, SWDs, NREM sleep and micro-arousals were automatically detected with the aid of computational algorithms based on the continuous wavelet transform. With this technical approach, we define the developmental changes of spike-wave epilepsy and sleep in WAG/Rij rats at the ages of 5, 7 and 9 months. The current study has the following aims: (1) to assess the developmental profile of spike-wave epilepsy in drug-naïve WAG/Rij rats; (2) to evaluate age-related changes of NREM sleep and micro-arousals during NREM sleep; (3) to test a hypothesis about the fragmentation of NREM sleep in epileptic WAG/Rij rats; (4) to analyze the associations between age-related changes of SWDs and changes of NREM sleep.

## 2. Materials and Methods

### 2.1. Animals

Experiments in rats were conducted at the Institute of Higher Nervous Activity and Neurophysiology RAS (Moscow, Russia) according to EU Directive 2010/63/EU for animal experiments and approved by our institution’s animal ethics committee. Rats were kept in standard conditions with a natural light/dark cycle and had free access to rat chow and tap water. Seventeen male rats were used in total.

### 2.2. EEG Recording

At the age of 4.5 months, rats were implanted with screw electrodes for epidural EEG recording (shaft length = 2.0 mm, head diameter = 2.0 mm, shaft diameter = 0.8 mm) over symmetrical right and left frontal cortex (AP +2 mm and L ± 2.5 mm) and occipital (AP −6 mm and L 4 mm). All coordinates are given relative to the bregma. The reference screw electrode was placed over the right cerebellum. Stereotactic surgery was performed under chloral hydrate anesthesia (325 mg/kg, 4% solution in 0.9% NaCl). Electrodes were permanently fixed to the skull with a methyl methacrylate monomer. Immediately after the surgery, rats received *i. m.* injections of metamizole (FSSCI Microgen, Russia, 25 mg/kg) for pain relief and then were housed in individual cages in order to prevent damages to electrode connectors and allowed to recover for a minimum of ten days before EEG recording. All animals underwent EEG recordings at the age of 5, 7 and 9 m in order to capture within-individual age-related changes.

After the surgery, rats were allowed to recover for a minimum of ten days. EEG recording was performed in freely moving rats placed in Plexiglas cages (25 × 60 × 60 cm) under 12:12 h light/dark cycle (light on at 8 a.m.). EEG signals fed into a multichannel amplifier (PowerLab 4/35, LabChart 8.0 software, ADInstruments, Sydney, Australia) via a swivel contact, band-pass filtered between 0.5–200 Hz, digitized with 400 samples/second/per channel and stored in the hard disk. The full-length EEG recording session lasted approximately 24 h (avg 22.2 h, min 20.0 h, max 26.6 h). Immediately prior to the full-length EEG recording, video-EEG was recorded for 1 h using video camera Genius eFace 1325R and video capture module for LabChart.

Wavelet-based EEG analysis was performed during the dark phase, because the variability of transitions around SWDs in WAG/Rij rats during dark phase was greater than during light phase [33]. This implies that disturbances of sleep control mechanisms were well pronounced during the dark phase. Analysis was done during two 3 h periods of light/dark cycle: at the beginning of dark phase 21:00–23:59 (“beginning of the dark phase”) and at the end of dark phase 3:00–5:59 (“end of the dark phase”).

Inclusion criterion: the presence of SWDs at the age of 9 m (*n* = 15 rats). Two rats without SWDs at all ages 5, 7 and 9 m (i.e., non-epileptic phenotype) were excluded from this study.

### 2.3. Vigilance States and SWDs in Video-EEG Recordings

**Figure 1:**
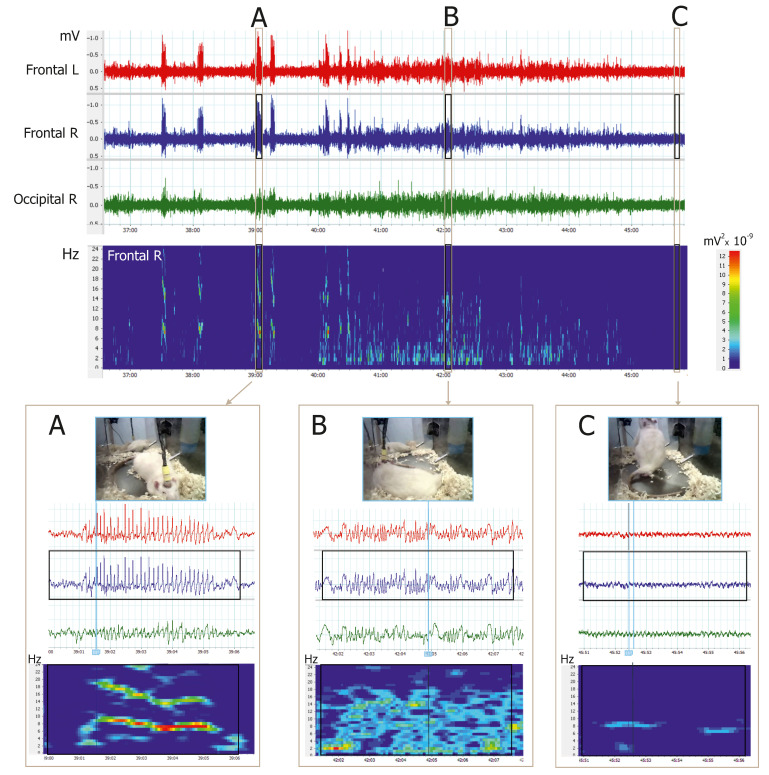
Examples of 3-channel video-EEG recordings in freely moving drug-naïve WAG/Rij rat (7 months old). Electrodes were implanted epidurally over the frontal left, frontal right and occipital right cortical areas. Time-frequency plot represents power spectra of the frontal right channel (FFT size = 512 data points, window overlap 93.75%, cosine-bell windowing). (**A**) Spontaneous spike-wave discharges (SWDs or spike-wave seizures) were seen in the frontal channels as bilaterally synchronous high-voltage repetitive spike-wave complexes with an intrinsic frequency of 8–10 Hz. (**B**) The state of NREM sleep was characterized by the presence of sleep spindles and delta waves in frontal and occipital channels. (**C**) The state of active wakefulness was characterized by desynchronized activity in frontal and occipital channels and theta activity associated with exploratory behavior.

One-hour video-EEG recordings were visually inspected in all subjects at the age of 9 months. Electrical brain activity was analyzed in accordance with principles of electrocorticographic analysis in rats [20]. Three stages were identified (Figure 1): (1) SWDs (spike-wave seizures)—in frontal channels: high-voltage bilaterally synchronous repetitive spike-wave complexes with sharp onset, >2 s duration and intrinsic frequency of 8–10 Hz (Figure 1A). (2) Slow-wave sleep (the state of NREM sleep)—in all channels: periods of >20 s duration with synchronized EEG containing sleep spindles and slow waves (Figure 1B). (3) Waking state—in all channels: periods of >20 s duration with desynchronized EEG with fast low-voltage activity, movement artifacts or theta rhythm associated with exploratory behavior (Figure 1C). REM sleep and the intermediate sleep states were disregarded.

### 2.4. Automatic Identification of Sleep/Waking States and SWDs in EEG

Behavioral sleep (BS) and waking state (AW) were detected using a wavelet-based algorithm, presented previously in Runnova, et al. (2021) [34]. To automatically detect the periods of SWDs, we performed additional calculations. As before, EEG signals from all three cortical channels underwent continuous wavelet transformation (CWT). EEG signals {*x*_1_, *x*_2_, *x*_3_} with duration *T* were recorded with a sampling frequency (1\*N*), i.e., a recording with a duration of 1 s contains *N* values. 

CWT *W*_i_(*f, t*) was calculated for each EEG signal *x_i_* based on the Morlet wavelet with the parameter Ω_0_ = 2π. With Ω_0_ = 2π, the time scale in CWT approximated to the classical representation of Fourier frequency *f*, Hz [35]. For automatic detection of SWDs and BS, it was empirically found that the maximum quality and detection speed were achieved when the analysis was performed in the frequency range Δ*f*_1_ = [2.5; 4.5], Δ*f*_2_ = [10.5; 12.5], Δ*f*_3_ = [15; 18], Δ*f*_4_ = [5; 10] Hz. For each EEG channel, we computed instantaneous CWT energy *E*_i_(*f, t*) as:

*E_i_*(*f*, *t*) = *W_i_* (*f*, *t*)^2^. (1)

The total instantaneous CWT energy *E*_Δ*f*1−4_(*t*) was calculated at each time point *t* in each frequency interval Δ*f*_1−4_ as:

(2) $$E_{\Delta f_{1-4}}^{i}\left(t\right)=\sum_{f\in \Delta f_{1-4}}^{}E_{i}\left(f,t\right).$$

Note that calculation of the instantaneous energy was limited to bands Δ*f*_1−4_; therefore, the number of operations and machine time required for the analysis of experimental data was substantially reduced. 

The integral value of the total instantaneous energy $E_{\Delta f_{j}}^{i}$ (2) in every band Δ*f*_1−4_ takes the form:

(3) $$\varepsilon_{\Delta f_{1-4}}^{i}\left(t_{0}\right)=N\cdot \Delta t\cdot \sum_{t_{1}}^{t_{2}}E_{\Delta f_{1-4}}^{i}\left(t\right),$$

where *t*_0_ is current time moment, Δ*t* = 0.5 s, *t*_1_ = t_0_ − 0.25·Δ*t*, and *t*_2_ = *t*_0_ + 0.25·Δ*t*.

**Figure 2:**
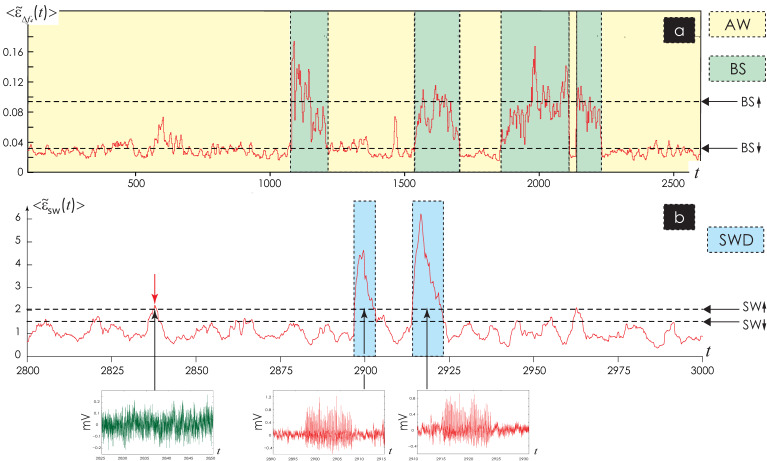
Details of the automatic detection of sleep and wake states (**a**) and spike-wave discharges, SWD (**b**) in 3-channel EEG data. (**a**) The fragment of timing dependence $\langle {\tilde{\varepsilon }}_{\Delta f_{4}}\left(t_{0}\right)\rangle$ as computed in EEG signals *x*_1_(*t*), *x*_2_(*t*), *x*_3_(*t*) in rat #1. The colored areas and the dashed vertical lines show detected states of behavioral sleep (BS, green) and wakefulness (AW, yellow). The horizontal thick dashed lines mark the threshold values BS↑ and BS↓. (**b**) The results of automatic detection of SWDs: a fragment of timing dependence $\;\langle {\tilde{\varepsilon }}_{sw}\left(t_{0}\right)\rangle$ as computed in EEG signals *x*_1_(*t*), *x*_2_(*t*), *x*_3_(*t*) in rat #2. The colored areas and the dashed vertical lines show detected SWDs (blue). The horizontal thick dashed lines mark the threshold values SW↑ and SW↓. Red arrowhead marks the artifact in which the energy characteristic $\;\langle {\tilde{\varepsilon }}_{sw}\left(t_{0}\right)\rangle$ reaches the threshold, but the criterion (15) is erroneous.

The identification of sleep\awake states is based on analysis of bioelectrical activity only in the band Δ*f*_4_, as we considered in detail in [34]. For all EEG channels, we assessed individual characteristics of EEG activity for each animal, namely, the integral multichannel energy characteristic $\langle {\tilde{\varepsilon }}_{\Delta f_{4}}\left(t_{0}\right)\rangle$, threshold values BS↑ and BS↓. The comparison of the integrated energy characteristic and threshold values is evaluated on two stages as shown in Figure 2a.

Detection of SWDs was based on energy characteristic of EEG measured in the bands Δ*f*_1−3_. The time dependence of the energy ratio energy ratio $\varepsilon_{sw}^{i}\left(t_{0}\right)$ was defined in each EEG channel as

(4) εswit0=εΔf3it0εΔf1it0+εΔf2it0.

For all EEG channels, we assessed the multichannel energy characteristic ${\tilde{\varepsilon }}_{sw}\left(t_{0}\right)$ as:

(5) ε˜swt0=∑i=13εswit03.

The integral value of energy characteristic ${\tilde{\varepsilon }}_{sw}\left(t_{0}\right)$ (5) was calculated at each time point *t*_0_ as:

(6) $$\;\langle {\tilde{\varepsilon }}_{sw}\left(t_{0}\right)\rangle =N\cdot \tau \cdot \sum_{\tau_{1}}^{\tau_{2}}{\tilde{\varepsilon }}_{sw}\left(t_{0}\right),$$

where *t*_0_ is current time moment, *τ* = 3 s, *τ*_1_ = t_0_ − 1.5 *τ*, and *τ*_2_ = *t*_0_ + 1. 5·*τ*.

Next, we considered two threshold values SW↑ and SW↓, individual for every animal, in the form of:

(7) $$\mathrm{SW}↑=1.75\cdot N\cdot T\cdot \sum_{0}^{T}\;\langle {\tilde{\varepsilon }}_{sw}\left(t_{0}\right)\rangle ,$$

(8) $$\mathrm{SW}↓=1.55\cdot N\cdot T\cdot \sum_{0}^{T}\;\langle {\tilde{\varepsilon }}_{sw}\left(t_{0}\right)\rangle .$$

The moment of SWD onset was detected at the time t↑, in which the value of $\;\langle {\tilde{\varepsilon }}_{sw}\left(t\right)\rangle$ (6) exceeded the threshold SW↑ (7), i.e., $\;\langle {\tilde{\varepsilon }}_{sw}\left(t↑\right)\rangle >SW↑$. Next, to detect the end moment of SWD, we compared the current value of the dependence (6) with the threshold value SW↓ (8), $\;\langle {\tilde{\varepsilon }}_{sw}\left(t↓\right)\rangle >SW↓$.

The time interval [t↑; t↓] corresponded to SWD as shown in Figure 2b. The variability of SWD durations even in one animal was large impeding selection of a reliable time threshold for assessing correctness of the interval [t↑; t↓]. In this case, the direct use of ratios (6) and (7), (8) could lead to false detection of oscillatory artifacts in EEG signals. To reduce the number of cases of erroneous detection, we proposed the special procedure. Firstly, the *extrema* (maxima {*max*_1_(*x*(*t*)); *max*_2_(*x*(*t*)); … *max_b_*(*x*(*t*))} and minimum {*min*_1_(*x*(*t*)); *min*_2_(*x*(*t*)); … *min_b_*(*x*(*t*))}) of the *x*(*t*) were defined in the time window Δ*T* with duration 5 s 1 s before the onset of SWD, i.e., Δ*T* = [t↑ − 6; t↑ − 5], and, secondly, the same extrema points ({*max*_1_(*x*(*t*)); *max*_2_(*x*(*t*)); … *max_swd_*(*x*(*t*))}, {*min*_1_(*x*(*t*)); *min*_2_(*x*(*t*)); … *min_swd_*(*x*(*t*))}) were detected during the identified time interval [t↑; t↓]. Thirdly, for each time interval [t↑; t↓], corresponding to SWD, we estimated the values *Xmax*, *Xmin* according to the following formulas:

(9) Xmax=∑i=1swdmaxixt, t∈t↑; t↓∑i=1mamaxixt, t∈t↑−6; t↓−5,

(10) Xmin=∑i=1swdminixt, t∈t↑; t↓∑i=1miminixt, t∈t↑−6; t↓−5.

Finally, in order to detect the correctness of SWD detection, we verified the validity of the following criterion

(*Xmax* + *Xmin*) > 6. (11)

**Figure 3:**
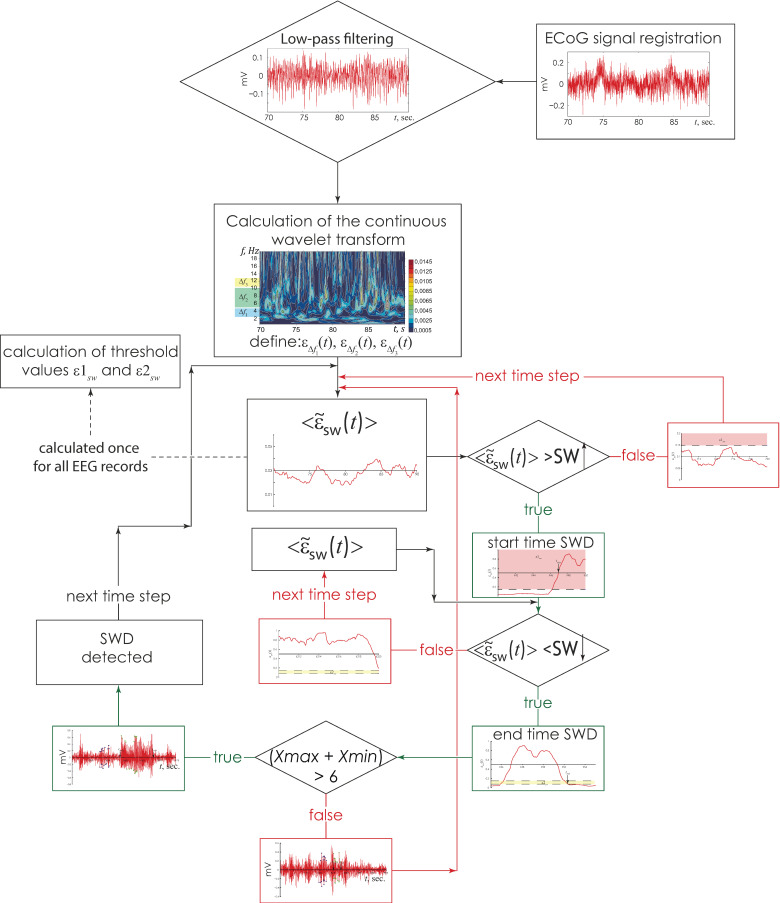
The diagram of the software implementation of the described method for multichannel registration EEG.

The SWD detection of time interval [t↑; t↓] was considered incorrectly defined if the criterion (15) was erroneous, i.e., (*Xmax* + *Xmin*) < 6, (Figure 2b, time interval highlighted red arrow). The main stages of the calculation of SWD detection method are presented in Figure 3.

Before independent use, a comparative statistical evaluation of the success of detecting various physiological states in animals was carried out based on manual processing of an hour-long video EEG by a neurophysiologist and the methods described. The average accuracy of automatic identification in assessing the duration of time intervals of different physiological states was achieved (i) 96.53% to detect durations of awake states, (ii) 94.70% for behavioral sleep and (iii) 97.34% for spontaneous SWD.

### 2.5. Identification of Micro-Arousals in EEG

Special attention was paid to brief episodes of waking during sleep, so-called micro-arousals. Micro-arousals were referred to as “*phasic EEG events which were not associated with awakenings regardless of their desynchronizational or synchronizational (sleep response-like) morphology and regardless of their connection with autonomic or some sort of behavioral arousal*” [36]. In accordance with the criteria developed by the Sleep Disorders Atlas Task Force of the American Academy of Sleep Medicine [37], we detected micro-arousals as 3–15 s waking state preceded by at least 10 s of noninterrupted sleep. Micro-arousals were detected automatically using the abovementioned approach (Section 2.4) and represented brief periods of wakefulness (up to 15 s) that were preceded and followed by BS; we have recently introduced this method in [34]. Wakening episodes longer than 15 s were recognized as wakefulness.

### 2.6. Statistical Analysis

Data are shown in the text as mean ± standard deviation. Data are shown in figures as median, 25–75% percentile and min–max. Statistical analysis was done using three-factor ANOVA (“Age”, “Period” and “Epi-phenotype”), in addition, repeated-measures ANOVA, Friedman’s ANOVA and Wilcoxon matched pairs test were used in selected groups. *p*-values less than 0.05 were reported as statistically significant.

## 3. Results

### 3.1. Age-Related Dynamics of SWDs

**Figure 4:**
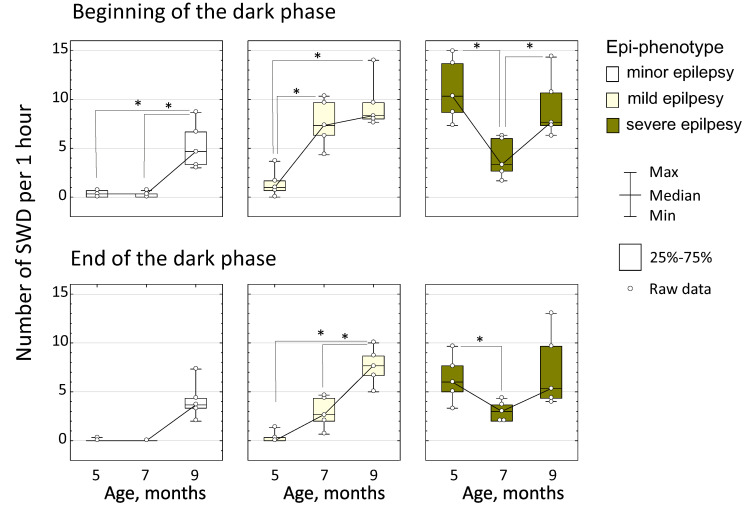
Age-related dynamics of the number of SWDs in rats with different epi-phenotypes. Asterisked are significant differences between ages according to the Wilcoxon test (*p* < 0.05).

The number and duration of SWDs highly varied across animals. Considering age-related changes in number of SWDs, we defined three epi-phenotypes: minor, mild and severe epilepsy. It was found that the factor “Epi-phenotype” significantly affected the number of SWDs at the beginning of the dark phase (F_2;36_ = 28.8, *p* < 0.0001) and at the end of the dark phase (F_2;36_ = 19.9, *p* < 0.0001, Figure 4). Interactions “Age” * “Epi-phenotype” were also significant (at the beginning of the dark phase—F_4;36_ = 11.7, *p* < 0.0001, and at the end of the dark phase—F_4;36_ = 4.3, *p* < 0.01), suggesting that age-related changes in the number of SWDs were different in different epi-phenotypes.

In rats with minor epilepsy (*n* = 5), SWDs were detected only at the age of 9 months (the mean number of SWDs was the same at the beginning and at the end of the dark phase, 5.3 ± 2.2 per h). An age-related increase in the number of SWDs was significant at the beginning of the dark phase (Friedman’s ANOVA, χ^2^ (2) = 7.89, *p* = 0.019) and at the end of the dark phase (Friedman’s ANOVA, χ^2^ (2) = 9.5, *p* = 0.009).

In rats with mild epilepsy (*n* = 5), Friedman’s ANOVA also indicated significant differences in the number of SWDs between three ages at the beginning of the dark phase (χ^2^ (2) = 7.6, *p* = 0.022) and at the end of the dark phase (χ^2^ (2) = 8.4, *p* = 0.015). These rats showed very few SWDs at the age of 5 months (0.9 ± 1.2 per h), and the number of SWDs increased to 5.2 ± 3.2 per h at the age of 7 m and to 9.5 ± 2.5 per h at the age of 9 m (all *p* < 0.05, Wilcoxon test).

In rats with severe epilepsy (*n* = 5), age-related differences in the number of SWDs were defined with Friedman’s ANOVA at the beginning of the dark phase (χ^2^ (2) = 7.6, *p* = 0.022) and at the end of the dark phase (χ^2^ (2) = 8.4, *p* = 0.015). These rats showed a temporal regress of SWDs: the number of SWDs at the age of 5 months was already as high as 8.7 ± 3.7 per h and reduced to 3.5 ± 1.6 SWDs per h at the age of 7 months, and then increased to 9.3 ± 3.1 SWDs per h at the age of 9 months (Figure 4, all *p* < 0.05, Wilcoxon test). In general, rats with minor and mild spike-wave epilepsy demonstrated a progressive increase in SWD numbers that has been widely acknowledged before [4,27,29], but rats with severe epilepsy demonstrated peculiar regressive-progressive changes in SWD numbers that has not been reported.

### 3.2. Slow-Wave Sleep

**Figure 5:**
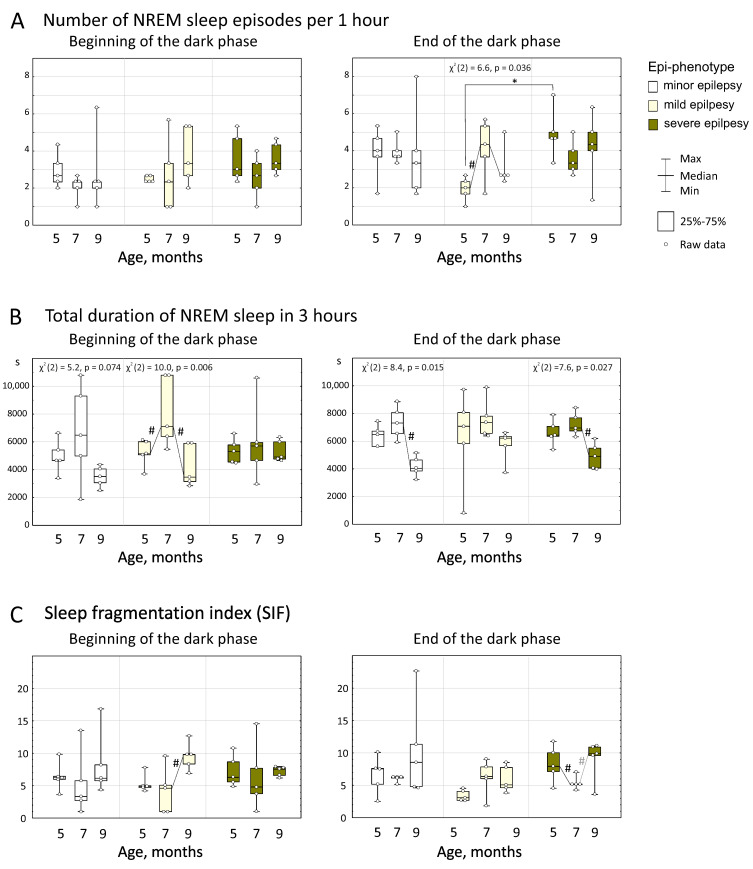
Characteristics of NREM sleep and sleep fragmentation (SFI) in WAG/Rij rats with different epi-phenotypes. Asterisked are significant differences between epi-phenotypes (Mann–Whitney test, *p* < 0.05). Chi-squares and *p*-values of Friedman’s ANOVA indicate a significant effect of the factor “Age” (#—significant differences according to Wilcoxon test, *p* < 0.05; a grey # indicated the tendency with *p* = 0.072).

Age-related changes in a number of NREM sleep episodes were analyzed with the repeated measures ANOVA (Figure 5A). The factors of “Age” and “Epi-phenotype” did not significantly affect the number of NREM sleep episodes (*p* > 0.05). The effect of the factor of “Period” was significant (F_1;24_ = 6.17, *p* = 0.020), suggesting that the number of NREM sleep episodes in the whole group (*n* = 15 rats) at the beginning of the dark phase was lower than at the end of the dark phase in each age: at the age of 5 months—3.0 ± 1.0 vs. 3.6 ± 1.7; at the age of 7 months—2.4 ± 1.3 vs. 3.9 ± 1.1 and at the age of 9 months—3.4 ± 1.5 vs. 3.7 ± 1.8. Friedman’s ANOVA indicated significant age-related differences in the number of NREM sleep episodes only in rats with mild epilepsy (*n* = 5 rats) at the end of the dark phase (χ^2^ (2) = 6.6, *p* = 0.036, Figure 5A). In these rats, the number of NREM sleep episodes at the age of 5 months was minimal (1.9 ± 0.6 per h) then increased to 4.1 ± 1.6 per h at the age of 7 months (*p* < 0.05, Wilcoxon test) and showed no significant changes at the age of 9 months (3.1 ± 1.1 per h). An increase in the number of NREM sleep episodes might be considered as a sign of NREM sleep fragmentation, which was found only in WAG/Rij rats with mild epilepsy between 5 and 7 months of age.

The total duration of NREM sleep was measured during 3 h epochs and statistically analyzed. The repeated measures ANOVA indicated a significant effect of the factor “Age” (F_2;48_ = 21.1, *p* < 0.005). More specifically, NREM sleep at the age of 5 months lasted in average 5801 ± 1631 s (i.e., approx. 1.6 h or 53% of the time of 3 h period) and at the age of 7 months its duration increased to 7148 ± 2175 s (*p* < 0.05, post hoc Bonferroni test) and at the age of 9 months reduced to 4651 ± 1204 s (*p* < 0.05, post hoc Bonferroni test). Neither the factor “Period”, nor “Epi-phenotype” affected the total duration of NREM sleep. With a significant effect of the “Age” factor on NREM sleep duration, it can be specified that the average percent of the automatically identified NREM sleep during the dark phase in WAG/Rij rats was 53% at the age of 5 months, 66%—7 months and 43%—9 months.

Figure 5B illustrates age-related changes in the total duration of NREM in different epi-phenotypes. In subjects with minor epilepsy, significant differences between ages were found at the end of the dark phase (Friedman’s ANOVA, χ^2^ (2) = 8.4, *p* = 0.015, Figure 5B). These rats showed the same duration of NREM sleep at the age of 5 and 7 months (6374 ± 771 s and 7301 ± 1168 s correspondingly) then a decrease to 4179 ± 741 s at the age of 9 months (*p* = 0.043, Wilcoxon test, Figure 5B). In these rats, a similar tendency was detected at the beginning of the dark phase (Friedman’s ANOVA, χ^2^ (2) = 5.2, *p* = 0.074, Figure 5B). In subjects with mild epilepsy, significant differences between ages were found at the beginning of the dark phase (χ^2^ (2) = 10.0, *p* = 0.006, Figure 5B), in particular, the duration of NREM sleep at the age of 5 months was 5208 ± 972 s and increased to 8111 ± 2519 s at the age of 7 months then reduced to 4259 ± 1525 s at the age of 9 months (both *p* = 0.043, Wilcoxon test). In subjects with severe epilepsy, significant differences between ages were found at the end of the dark phase (χ^2^ (2) = 7.6, *p* = 0.027, Figure 5B). In these rats, duration of NREM sleep at the age of 5 and at the age of 7 months did not differ (6631 ± 936 s and 7219 ± 830 s, correspondingly) and then increased to 4924 ± 944 s at the age of 9 months (*p* = 0.043, Wilcoxon test). In summary, a reduction of NREM sleep duration between 7 and 9 months of age was found in all WAG/Rij rats, but during different time periods: in subjects minor and severe epilepsy—at the end of the dark phase; and in subjects with mild epilepsy—at the beginning of the dark phase. It is remarkable that the number of SWDs increased between the age of 7 and 9 months in all epi-phenotypes (see above, Figure 4) in parallel with the shortening of the total time of NREM sleep.

In order to better evaluate sleep characteristics, a sleep fragmentation index (SFI) was calculated as the number of sleep episodes divided by the total duration of sleep in hours. It was found that SFI significantly changed with age (“Age” factor F_2;48_ = 7.4, *p* < 0.005), but it was not affected by factors “Period” and “Epi-phenotype”. Post hoc Bonferroni test for the factor “Age” indicated that the value of SFI at the age of 5 and 7 months did not differ (6.3 ± 2.6 and 5.6 ± 3.2 correspondingly, *p* > 0.05), but it increased to 8.4 ± 4.0 at the age of 9 months (data not shown). Therefore the period 7–9 months of age is characterized by a progressive fragmentation of sleep in all rats. This effect was pronounced in subjects with mild epilepsy at the beginning of the dark phase (#—significant increase of SFI in Figure 5C), and the same tendency was found in rats with severe epilepsy at the end of the dark phase (*p* = 0.072).

### 3.3. Micro-Arousals

**Figure 6:**
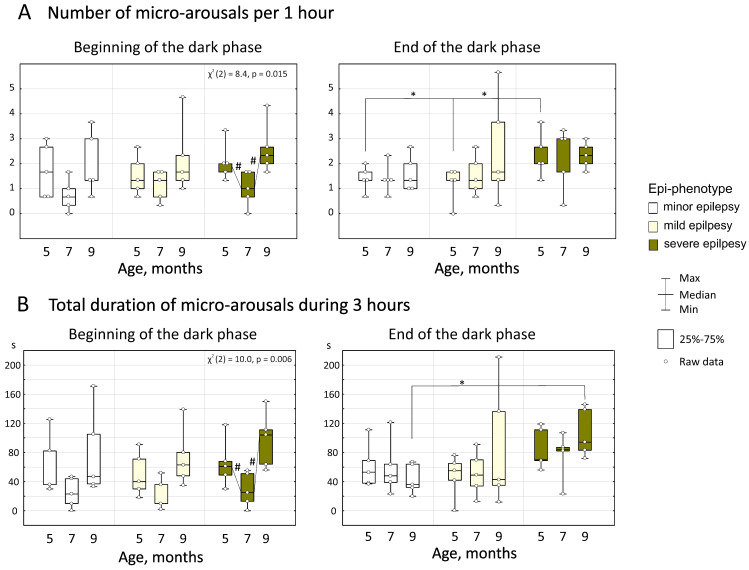
Characteristics of micro-arousals in WAG/Rij rats with different epi-phenotypes. Asterisked are significant differences between epi-phenotypes (Mann–Whitney test, *p* < 0.05). Chi-squares and *p*-values of Friedman’s ANOVA indicate a significant effect of the factor “Age” (#—significant differences according to Wilcoxon test, *p* < 0.05).

The number of micro-arousals changed with age (repeated measures ANOVA, factor “Age” F_2;48_ = 7.7, *p* < 0.005). Friedman’s ANOVA displayed significant age-related changes in the number of micro-arousals only in rats with severe epilepsy at the beginning of the dark phase (χ^2^ (2) = 8.4, *p* = 0.015, Figure 6A). In these rats, the number of micro-arousals at the age of 5 months was 2.1 ± 0.8 per h and reduced to 1.0 ± 0.7 per h at the age of 7 months, and then increased to 2.6 ± 1.0 per h at the age of 9 months (all *p* < 0.05, Wilcoxon test, Figure 6A). At the age of 5 months, rats with severe epilepsy demonstrated a higher number of micro-arousals at the end of the dark phase (2.3 ± 0.9 per h) in comparison to rats with minor and mild epilepsy (1.4 ± 0.5 and 1.2 ± 0.7 per h correspondingly, all *p* < 0.05, Wilcoxon test, Figure 6A).

Furthermore, the total duration of micro-arousals in 3 h changed with age (repeated measures ANOVA, factor “Age” F_2;48_ = 9.4, *p* < 0.00034). Significant age-related changes in the total duration of micro-arousals were found only in rats with severe epilepsy at the beginning of the dark phase (Friedman’s ANOVA, χ^2^ (2) = 8.4, *p* = 0.015, Figure 6B). In these rats, the total duration of micro-arousals at the age of 5 months was 65.2 ± 32.8 s, and it reduced to 28.8 ± 23.8 s at the age of 7 months and then increased to 97.0 ± 38.2 at the age of 9 months (all *p* < 0.05, Wilcoxon test, Figure 6B). At the age of 9 months, rats with severe epilepsy displayed a longer total duration of micro-arousals at the end of the dark phase (106.8 ± 33.6 s) in comparison to rats with minor epilepsy (43.8 ± 20.7 s, *p* < 0.05, Mann–Whitney test, Figure 6B).

### 3.4. Relationship between SWDs, NREM Sleep and Micro-Arousals

**Figure 7:**
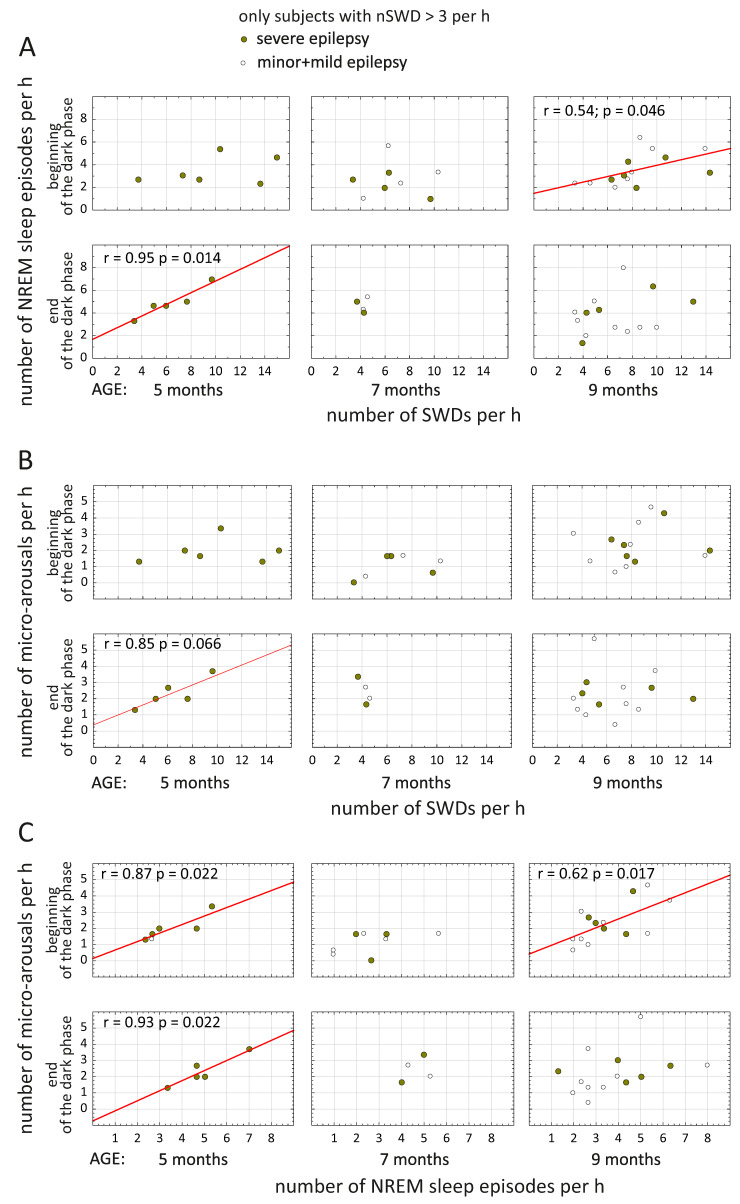
Pearson’s correlations between the number of SWDs, number of NREM sleep episodes and number of micro-arousals during NREM sleep. Significant correlations are indicated with red lines and r values.

Analysis of Pearson’s correlations was performed in rats who expressed more than three SWDs per hour. A strong positive correlation between the number of SWDs and the number of NREM sleep episodes was found at the age of 5 months only in subjects with severe epilepsy (r = 0.95, *p* = 0.014 at the end of the dark phase, Figure 7A). A similar, but less pronounced correlation was found at the age of 9 months in all subjects (r = 0.54, *p* = 0.046 at the beginning of the dark phase, Figure 7A). The duration of the SWDs did not significantly correlate with the duration of NREM sleep episodes. In short, the higher the incidence of SWDs, the higher the number of NREM sleep episodes, and this might be interpreted as fragmentation of NREM sleep.

The number of SWDs showed a strong tendency to correlate with the number of micro-arousals only in subjects with severe epilepsy at the age of 5 months (r = 0.85, *p* = 0.066 at the end of the dark phase, Figure 7B), suggesting the higher incidence of SWDs in subjects with more frequent micro-arousals. This link seemed peculiar and was further investigated (see below). The duration of SWDs did not significantly correlate with the duration of micro-arousals. Meanwhile, the number of micro-arousals showed a positive correlation with the number of NREM sleep episodes, again in subjects with severe epilepsy at the age of 5 months (r = 0.93, *p* = 0.022 at the end of the dark phase; r = 0.87, *p* = 0.022 at the beginning of the dark phase, Figure 7C). The same correlations were found at the age of 9 months in all subjects (r = 0.62, *p* = 0.017 at the beginning of the dark phase, Figure 7C). Correlations between the number of NREM sleep episodes and the number of micro-arousals (Figure 7C) might result from a common mechanism of arousal control.

**Figure 8:**
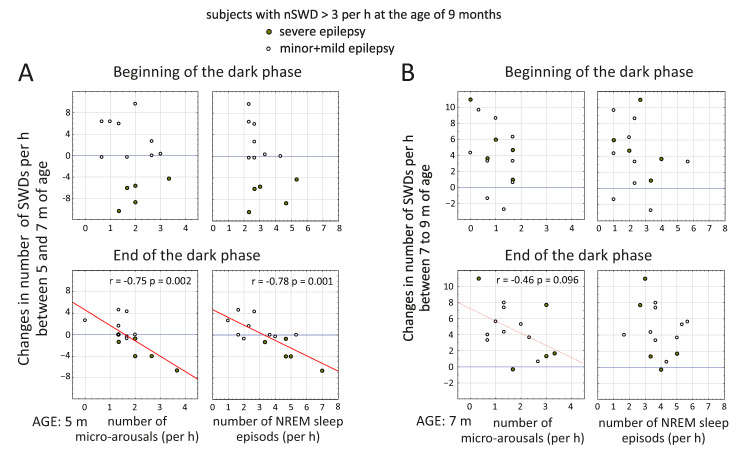
Pearson’s correlations between age-related changes in SWDs and the number of NREM sleep episodes/micro-arousals in WAG/Rij rats. Significant correlations are indicated with red lines and r values.

In order to examine a prognostic value of micro-arousals in spike-wave epilepsy, we computed the difference in number/duration of SWDs between the age of 5 and 7 months and the age of 7 and 9 months in each rat. Rats with severe epilepsy (*n* = 5) showed a decrease in the number of SWDs between the ages of 5 and 7 months (negative values in Figure 8A). It was surprising that the difference in the number of SWDs between 5 to 7 months of age showed strong negative correlations with the number of micro-arousals at the age of 5 months (r = −0.75, *p* = 0.002 at the end of the dark phase, Figure 8A) and with the number of NREM sleep episodes at the age of 5 months (r = −0.78, *p* = 0.001, also at the end of the dark phase, Figure 8A). Therefore, WAG/Rij rats with a higher number of micro-arousals (and NREM sleep episodes) at the age of 5 months were characterized by a reduction of SWDs between 5 and 7 months of age.

The same analysis was performed to examine the relevance of micro-arousals and NREM sleep in changes of SWDs between 7 and 9 months of age (Figure 8B). The number of micro-arousals at the age of 7 months showed just a tendency for negative correlations with the difference in SWD numbers between 7 and 9 months the age (r = −0.46, *p* = 0.096 at the end of the dark phase, Figure 8B). Therefore, micro-arousals and NREM sleep in 7-month-old WAG/Rij rats were not associated with an increase in SWDs in advancing ages.

## 4. Discussion

Here we performed an automatic analysis of three-channel EEGs recorded in freely moving drug naïve WAG/Rij rats repeatedly at the ages of 5, 7 and 9 months. We divided the experimental cohort of animals into three groups according to the severity of epileptic activity. In contrast to the Genetic Absence Epilepsy Rat from Strasbourg, GAERS rats, which are also used as a validated model of absence epilepsy and have a control strain of non-epileptic animals (NEC) derived from the same ancestor, WAG/Rij rats have no genetically close control. We solved this problem at Moscow’s Institute of Higher Nervous Activity by breeding non-epileptic WAG/Rij rats, and using them as a control subgroup of WAG/Rij rats that do not show pronounced spike-wave activity. In the total population of WAG/Rij rats at our Institute, the number of non-epileptic subjects reaches 25%. In the present study, only two rats did not show SWDs, and this number was too small to put them in a separate group, so they were removed from the analysis. The severity of spike-wave epilepsy in the remaining 15 rats was different, and, considering the age-related dynamics of SWDs, we divided them into three epi-phenotypes. This approach helped us to define the relationship between the development of SWDs and sleep fragmentation. We assumed that rats with more pronounced spike-wave activity had more fragmented slow-wave sleep, resembling patients with juvenile myoclonic epilepsy characterized by sleep instability when epileptiform discharges breaking through the state of reduced arousal (phase B of CAP) because of increased epileptic pressure [38].

### 4.1. Automatic Analysis of Three-Channel EEG Data

Here we introduced a new methodological approach to automatically analyze multichannel EEG data obtained in freely moving rats. First, we used a wavelet-based method of automatic sleep recognition. We found that an increased wavelet power in the 5–10 Hz frequency band as measured in frontal and occipital cortical areas could be used as a reliable marker of behavioral sleep (as detected manually). This can account for neuronal synchronization and the presence of so-called mid-frequency oscillations such as the sleep spindles and 5–9 Hz oscillations detected in WAG/Rij rats [7,39]. Secondly, we proposed the possibility of a parallel use of automatic identification of spike-wave seizures. The number of required numerical operations in the method was reduced about 1.5 times in comparison with the method described earlier in [40]. The method of SWDs detection was fully automated, and all individual parameters for every animal were calculated without the manual involvement of an experienced neurophysiologist.

The proposed methods for automatic EEG analysis can be used in experimental neurophysiology to study and diagnose the physiological and pathological activity of brain activity in rats. Accurate identification of normal physiological and pathological conditions in laboratory animals is an important task of this research area, since brain activity changes with the chronic development of diseases, in particular, with the development of such neurological disorders as epilepsy.

Objective diagnostics of the moments of falling asleep, awakening, the beginning and end of periods of epileptic activity allow us to investigate subtle changes in the characteristics of brain activity that precede or occur in various physiological states of animals, for example, as in studies [41,42]. Moreover, such developments can be used as the basis of devices for monitoring the human operators’ wakefulness.

The number of methods for automatic identification of some specific oscillatory phases in the brain biopotentials, such as SWD, spindle and ripple activity has increased in recent years. However, various types of spindle patterns have been characterized by a large variability even within the same organism [43]. The robust detection of corresponding patterns in EEG primarily has been provided by the use of machine learning, ANN and statistical models methods. In particular, Kulkarni et al. [44] demonstrated a deep learning strategy to identify sleep spindles from a single recording of human brain activity in real time with a latency of up to 350 ms. All the same, the key problem with the use of machine learning methods is the impossibility of considering dynamics scenarios for a certain oscillatory activity. From this point of view, time-frequency analysis is preferable and gives a clue to many aspects of EEG oscillations, for instance, the study of stable precursors and characteristic features of sleep-related specific oscillatory events in EEG. An interdisciplinary team led by Evgenia Sitnikova has been working in this area of research for more than a decade, and in particular, performing comprehensive studies of spike-wave seizures and sleep spindles in WAG/Rij rats (for example, [45,46,47], etc.).

At the same time, detection of the physiological states (sleep, wakefulness) in laboratory animals could be done either by visual analysis of video-EEG records [7] or automatically using additional information about muscle activity and/or oculomotor activity [48,49,50]. Here, we presented a method for the reliable detection of NREM sleep and wakefulness with high specificity and accuracy based on the analysis only of multichannel recordings of electrical brain activity. In addition, this automatic system has a natural adaptation to analyze the sleep fragmentation or the periods and frequency of micro-arousals.

### 4.2. Spike-Wave Epilepsy in WAG/Rij Rats: Epi-Phenotypes and Characteristics of NREM Sleep

Here, we found that the number of SWDs in WAG/Rij rats fluctuated from 0 to 43 SWDs per 3 h, suggesting a notable phenotypic heterogeneity of spike-wave epilepsy. We scored the number of SWDs and assessed age-related changes in order to better characterize epileptic phenotypes. Three epi-phenotypes in WAG/Rij rats were defined based on the number of SWDs: minor, mild and severe epilepsy. In particular, rats with minor spike-wave epilepsy showed epileptic EEG activity only at the age of 9 months (~5 SWDs per h). Rats with mild spike-wave epilepsy characterized by insignificant epileptic activity at the age of 5 months; though the number of SWDs at the age of 7 months was ~5 per h and increased to ~10 SWDs per h at the age of 9 months. An age-related increase in SWD numbers in WAG/Rij rats was acknowledged before [28,30], but peculiar age-related changes of spike-wave activity were found in rats with severe spike-wave epilepsy (5 out of 15 subjects). These subjects showed retrograde dynamics of SWD numbers between 5 and 7 months: ~9 and ~4 SWDs per h correspondingly, whereas SWD numbers returned to ~9 SWDs per h at the age of 9 months. 

The decrease in the number of SWDs in WAG/Rij rats with severe epilepsy between 5 and 7 months of age contradicts the existing knowledge and requires special attention. Age-related distributions of SWDs and other analyzed parameters showed a similar V-shaped pattern. This might be caused by a change in the general condition of animals with severe epileptic activity during recording at 7 months compared to recordings at 5 and 9 months. Based on the data we have, we cannot draw a conclusion about the influence of any external factor that led to this difference. All rats were born during the same period (November–December) of one year. Accordingly, recordings at 5 months were made in April–May, at 7 months in June–July, at 9 months in August–September. All rats were kept in the same vivarium and were recorded in the same experimental room. The presence of seasonal activity cycles in laboratory animals is a matter of debate [51]. Assuming seasonal fluctuations in activity in laboratory rats, it can be proposed that they specifically affected rats from the group with severe epilepsy and led to the appearance of a V-shaped pattern.

Another interesting finding relates to the age-related dynamics of NREM sleep in WAG/Rij arts. A reduction of NREM sleep duration between 7 and 9 months of age was found in all epi-phenotypes, and the factor “Epi-phenotype” was not significant. It is remarkable that the number of SWDs increased between the age of 7 and 9 months in all epi-phenotypes in parallel with the shortening of the total time of NREM sleep. This stresses the relationship between spike-wave epilepsy and fragmentation of NREM sleep in WAG/Rij rats [31] and supports the idea of Peter Halász et al. [21]: *“not only sleep instability promotes the appearance of SWD, but SWD may also cause instability”.*

Current results provide further evidence that spike-wave epilepsy in WAG/Rij rats was associated with fragmentation of NREM. A growing number of NREM sleep episodes in rats with mild epilepsy between the age of 5 and 7 months (Figure 5A) might be considered as a sign of NREM sleep fragmentation coinciding with a growing number of SWDs. In addition, an increase in the number of SWDs between the ages of 7 and 9 months characterizing all epi-phenotypes (Figure 2) appeared in parallel with shortening of the total time of NREM sleep. This fits well to an increase of the sleep fragmentation index between 7 and 9 months of age (all groups mean: SFI elevation from 5.6 to 8.4). Finally, the number of SWDs positively correlated with the number of NREM sleep episodes that was more noticeable in rats with severe epilepsy already at the age of 5 months and in all rats at the age of 9 months (Figure 5A).

### 4.3. Spike-Wave Epilepsy and Micro-Arousals during NREM Sleep

Our data indicated that spike-wave epilepsy per se seems to promote micro-arousals during NREM sleep. More specifically, we found that rats with severe epilepsy (1) displayed a higher number of micro-arousals than rats with minor and mild epilepsy at the age of 9 months (Figure 6A); (2) displayed a longer total duration of micro-arousals than rats with minor epilepsy at the age of 9 months (Figure 6B); (3) showed a tendency to positive correlation between the number of SWDs and the number of micro-arousals at the age of 5 months. It is worth noting that subjects with severe epilepsy at the age of 5 months and all rats at the age of 9 months showed positive correlations between the number of micro-arousals and the number of NREM sleep episodes: the higher the number of micro-arousals, the higher the number of NREM sleep episodes. These associations might suggest a common mechanism controlling long and short arousals in WAG/Rij rats.

Here, we found peculiar negative correlations between the number of micro-arousals (and NREM sleep episodes) at the age of 5 months in rats and changes in the number of SWDs between 5 and 7 months of age. In particular, rats with severe epilepsy who had the most frequent micro-arousals (and NREM sleep episodes) at the age of 5 months, showed a reduction of spike-wave seizures between 5 and 7 months of age (Figure 6A). A higher number of micro-arousals in parallel with the higher number of NREM sleep episodes at the early symptomatic age (5 months) might play a safeguarding function, attenuating spike-wave epilepsy. Our results are in agreement with the opinion of [8]: “*Microarousals used to be considered harmful perturbations destabilizing sleep; however, they turned out to have a sleep-regulatory and a safeguarding function*”. In our WAG/Rij rats, the >“*safeguarding function*” of micro-arousals was only seen at the early symptomatic age (5 months). At the age of 7 months, micro-arousals and NREM sleep were not associated with an increase in SWDs at the age of 9 months.

There are two main wake-promoting brain pathways (ventral and dorsal) (reviewed in [52]). The dorsal pathway innervates the thalamus and the ventral pathway consists of fibers of the medial forebrain bundle, which pass through the midbrain, posterior/lateral hypothalamus, and basal forebrain. For full wakefulness, the activation of both pathways is needed, while during micro-arousals, only one of the pathways may be activated. It can be proposed that a greater number of micro-arousals reflects increased excitability of the dorsal wake-promoting pathway in animals with severe spike-wave activity. Such increased excitability can be induced by epileptogenesis, which creates a hypersynchronous thalamocortical network [53]. At the same time, micro-arousals are favorable for the occurrence of a spike-wave discharge [26]. Thus a vicious circle is formed: the development of SWD-fragmented sleep, and sleep fragmentation provokes SWDs.

Correlations between the number of NREM sleep episodes and the number of micro-arousals (Figure 5C) might imply a common mechanism of arousal control. The age-related increase of spike-wave epilepsy might be associated with impairment of sleep- and wake-promoting mechanisms [8,23,54] and the transformation of micro-arousals into arousals. This might lead to a higher sleep fragmentation in advancing age (9 months old).

## 5. Conclusions

Here we identified developmental profiles of three epi-phenotypes of spike-wave epilepsy in drug-naïve WAG/Rij rats at the ages of 5, 7 and 9 months: severe, mild and minor epilepsy. These epi-phenotypes appear to be distinctive, as long as rats with minor and mild epilepsy demonstrated a progressive increase in SWD numbers that has been acknowledged before [28,30], but rats with severe epilepsy demonstrated peculiar regressive–progressive changes in SWD numbers that have not been reported.

The age-related increase of spike-wave epilepsy in WAG/Rij rats was linked to the fragmentation of NREM sleep. Spike-wave epilepsy per se seems to promote micro-arousals during NREM sleep. We found a surprising indicator of different age-related dynamics of epileptic activity and sleep fragmentation in rats with varying severity of spike-wave epilepsy. WAG/Rij rats with a higher number of micro-arousals (and NREM sleep episodes) at the age of 5 months were characterized by a reduction of SWDs between 5 and 7 months of age, but not in more advanced ages.

Rats with severe epilepsy had already developed numerous SWDs at the age of 5 months, and epileptogenic processes in their thalamocortical network might be associated with an increased excitability of the wake-promoting pathway and NREM sleep fragmentation. Rats with mild epilepsy enter the same stage only at the age of 9 months.

## Data Availability

The data that support the findings of this study are available from the corresponding author upon reasonable request.
